# CT findings of 795 COVID-19 positive cases: a multicenter study in Egypt

**DOI:** 10.1186/s43055-020-00351-7

**Published:** 2020-11-27

**Authors:** Youssriah Yahia Sabri, Mohamed Mohsen Tolba Fawzi, Eman Zaki Nossair, Safaa Mohamed El-Mandooh, Amira Aly Hegazy, Sally Fouad Tadros

**Affiliations:** 1 Kasr Al-Ainy Faculty of Medicine, Cairo University, Al-Manial, Cairo, 11559 Egypt; 2 National Hepatology & Tropical Medicine Research Institute (NHTMRI), 10 (A) Kasr El-Aini St, Cairo, 11796 Egypt

**Keywords:** COVID-19 ;Chest CT, Egypt

## Abstract

**Background:**

Corona Virus Disease 2019 (COVID-19) outbreak was officially announced as a global pandemic by the WHO on March 11^th^ 2020. Thorough understanding of CT imaging features of COVID-19 is essential for effective patient management; rationalizing the need for relevant research. The aim of this study was to analyze the chest CT findings of patients with real-time polymerase chain reaction (RT-PCR) proved COVID-19 admitted to four Egyptian hospitals. The recently published RSNA expert consensus statement on reporting COVID-19 chest CT findings was taken into consideration.

**Results:**

Normal CT “negative for COVID-19” was reported in 26.1% of our RT-PCR proved COVID-19 cases. In descending order of prevalence, imaging findings of the positive CT studies (73.9%) included GGO (69%), consolidation (49.7%), crazy paving (15.4%), and peri-lobular fibrosis (40.6%). These showed a dominantly bilateral (68.2%), peripheral (72.4%), and patchy (64.7%) distribution. Remarkably, thymic hyperplasia was identified in 14.3% of studies. According to the RSNA consensus, CT findings were classified as typical in 68.9%, indeterminate in 3.6%, and atypical in 1.4% of the evaluated CT studies.

**Conclusion:**

Although COVID-19 cannot be entirely excluded by chest CT, it can be distinguished in more than two-thirds of cases; making CT a widely available, non-invasive, and rapid diagnostic tool.

## Background

Coronavirus Disease 2019 (COVID-19) outbreak caused by “severe acute respiratory syndrome coronavirus 2” (SARS-CoV-2) was officially announced as a global pandemic by the World Health Organization (WHO) on March 11, 2020 [1, 2]*.* A total of 7,553,182 cases and 423,349 deaths had been reported worldwide by June 13, 2020, from which 41,303 cases and 1,422 deaths recorded in Egypt [3].

COVID-19 infection may be asymptomatic, may present with mild non-specific symptoms such as fever, cough, or fatigue, and may progress to severe symptoms including respiratory failure or even death in patients of old age and/or other co-morbidities [4].

The gold standard for diagnosing COVID-19 up till now is a positive nucleic acid testing (NAT) using reverse-transcriptase polymerase-chain-reaction (RT-PCR) [1]. Several papers reported the typical COVID-19 chest findings as multifocal bilateral ground glass opacities (GGOs) with or without patchy consolidations in a peripheral subpleural distribution and posterior lower lobe predilection [4]. However, further studies found other findings including crazy paving pattern, airway changes, reversed halo sign, etc. [5]. CT is more efficient in detection of GGO than radiography [4]. Thus various studies advocated the use of chest CT in the management of COVID-19 patients [2, 6, 7].

Four categories were proposed by the Radiological Society of North America (RSNA) Expert Consensus Statement on reporting chest CT Findings related to COVID-19 depending on the type of lesion encountered: (1) typical features which are observed frequently and more distinctly in COVID-19 pneumonia, (2) indeterminate features which are observed in COVID-19 pneumonia but are not characteristic, (3) atypical features which are infrequently observed in COVID-19 pneumonia and are more definitive of other infections, and (4) negative for pneumonia with no lung findings linked to infection, precisely, GGO and consolidation [2]. However, it should be recognized that chest CT may be negative early in COVID-19 [2].

The aim of this study was to analyze the chest CT findings of RT-PCR proved COVID-19 patients adopting the classification recommended by RSNA Expert Consensus Statement in order to test its diagnostic competence.

At that time, the health system in Egypt was still capable of isolating all PCR-confirmed cases of COVID-19 regardless of disease severity including asymptomatic contacts; to limit infection spread in the context of that phase of the pandemic [8].

## Methods

### Study population and design

Before conducting this prospective study, local institutional review board (IRB) approval was granted. Written informed consent was obtained from all study participants or their authorized representatives.

**Fig. 1:**
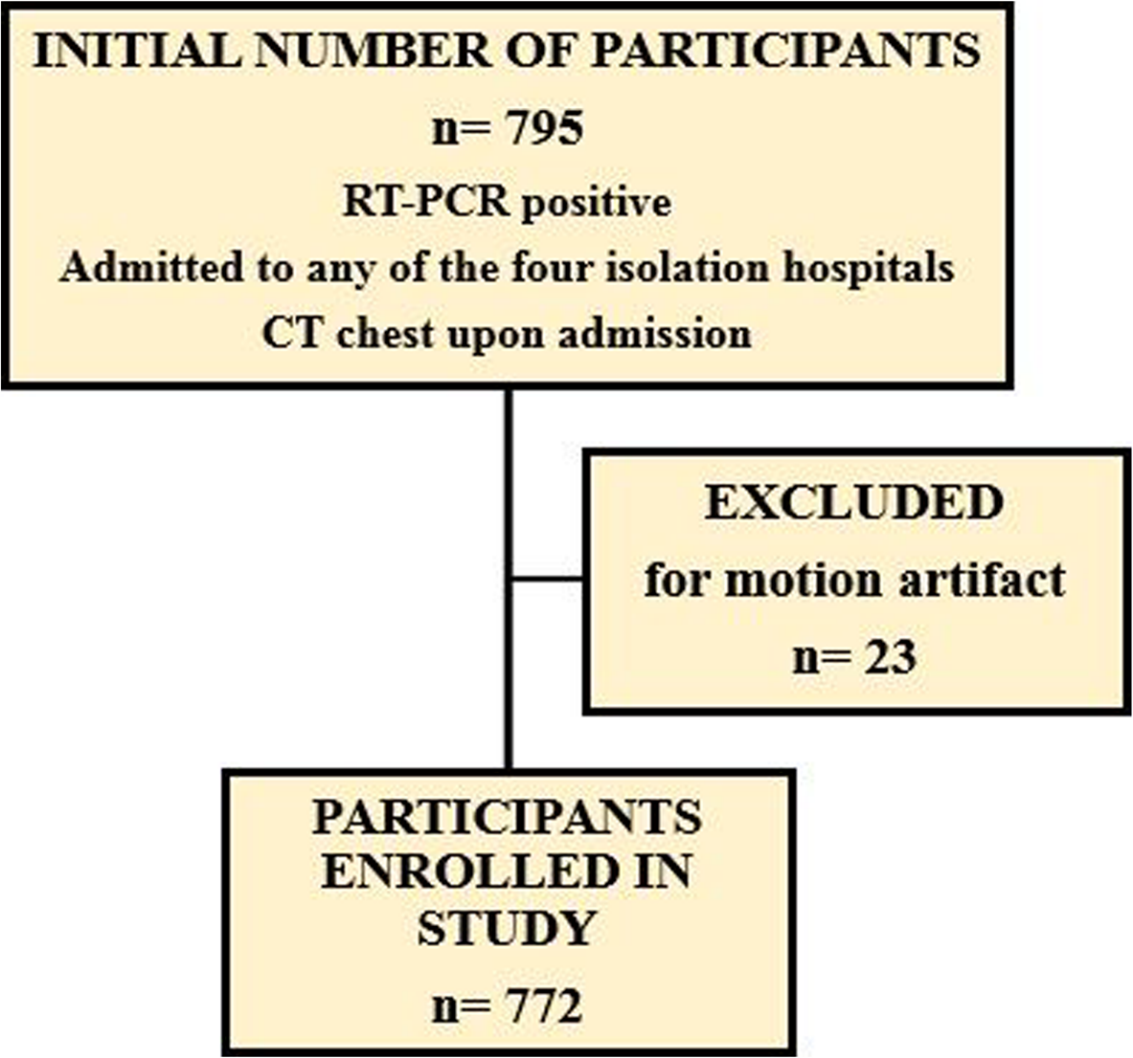
Study flow chart

This study initially involved 795 consecutive participants admitted to four Egyptian hospitals in the period from March 2, 2020 to June 13, 2020. All subjects had RT-PCR confirmed COVID-19 and underwent chest CT upon admission. Only those whose CT was degraded by motion artifacts were excluded (*n* = 23). Thereby, a total of 772 participants were enrolled in the study [431 males (age range, 4 months–95 years; mean, 45.66 years) and 341 females (age range, 3–95 years; mean, 44.11 years)] (Fig. 1).

All relevant clinical, laboratory, and epidemiologic data were provided by the admitting physicians.

### Imaging technique/acquisition

Participants were scanned with the following scanners: Aquilion Lightning™ “16-row 32 slice” (Toshiba Medical Systems) and Aquilion Prime™ “80-row 160 slice” (Toshiba Medical Systems).

Acquisition parameters were set at 120 kVp; 100–200 mAs; pitch, 0.75–1.5; and collimation, 0.625–5 mm. All imaging data were reconstructed using a medium sharp reconstruction algorithm with a slice thickness of 0.625–5 mm.

With the patient in supine position, CT images were acquired at full inspiration from the level of the thoracic inlet to the diaphragm. No IV contrast was administered.

### Imaging analysis

The performed studies were transferred to PACS system (PaxeraUltima version 6.0.0.1 and MILLENSYS version 6.5.0.2579) for reviewing. Five radiologists with 9 to 32 years of experience (Y.Y.S., M.F.T, E.Z.N, S.M.E. and S.F.T.) interpreted the CT studies. Each study was reviewed by two of the five radiologists independently. In case of discrepancy, studies were re-reviewed by the thoracic radiologist with 32 years of experience (Y.Y.S.) then findings were discussed to reach a general agreement. Scans were viewed in both lung (WW/WL: 1500/− 600 HU) and mediastinal (WW/WL 300/50 HU) windows.

Readers reported the presence of the following lung parenchymal findings: GGO, consolidation, crazy paving, peri-lobular fibrosis, reversed halo sign, vacuolar sign, pulmonary nodules, lobar pneumonia, and lung cavitation as well as associated traction bronchiectasis and vascular thickening. The involved lung lobes and lesions’ pattern of distribution whether peripheral, peripheral and central, patchy, or diffuse were recorded. Subsequently, CT findings were allocated to one of the RSNA consensus categories. Other associated pulmonary, mediastinal, pleural, and upper abdominal CT findings were registered as well.

Seventy-one participants underwent follow-up CT studies to evaluate the progression or regression of the aforementioned findings in addition to newly developed observations—their scans were assessed accordingly.

### Statistical analysis

The collected data were carefully revised, coded, tabulated, and introduced to a personal computer using “Microsoft Office Excel Software” program (2016) for windows by A.A.H. The pre-coded data were then transferred to the Statistical Package of Social Science Software program, version 23 to be statistically analyzed.

For qualitative variables, they were described as frequency and percentage. Comparison for qualitative variables was done by using chi-square test and Fisher’s exact test, where *p* value of significant correlation if *p* < 0.05.

Quantitative variables were presented using mean ± standard deviation (SD). Comparison between groups was performed using independent *t* test and analysis of variance (ANOVA) test followed by Bonferroni comparisons test. *P* values less than 0.05 were considered statistically significant.

## Results

This study enrolled 772 consecutive study participants [431 males and 341 females, age range 4 months–95 years, mean age 44.9 ± 16.2 years, inter-quartile range 32–58 (26) years]. All participants presented to one of the four hospitals with positive RT-PCR tests. The patients’ demographics and clinical data are listed in Table 1.

**Table 1:** Patients’ demographics and clinical data

	No. of patients (%)
Patients’ demographics:	
Number of CT studies	862
Number of patients	772
Mean age (years)	44.98 ± 16.21
Age range	4 months—95 years
Interquartile range (years)	32-58 (26)
Males	431 (55.8%)
Females	341 (44.2%)
Clinical symptoms	
Fever	581 (75.3%)
Cough	466 (60.4%)
Dyspnea	274 (35.5%)
Asymptomatic contacts	97 (12.6%)

A total of 862 CT chest studies were performed including 772 initial and 90 follow up CT studies. On initial CT study, 569/772 (73.7%) (age range 3 months—95 years; mean 49.84 years) had positive CT imaging manifestations of COVID-19, while 203/772 (26.3%) (age range 3—95 years; mean 31.33 years) did not show any lung abnormalities despite a positive RT-PCR test. There was a statistically significant difference regarding age between those with positive and negative CT studies; where the ones with positive CT were older than the ones with negative CT studies (*p* value < 0.001).

**Fig. 2:**
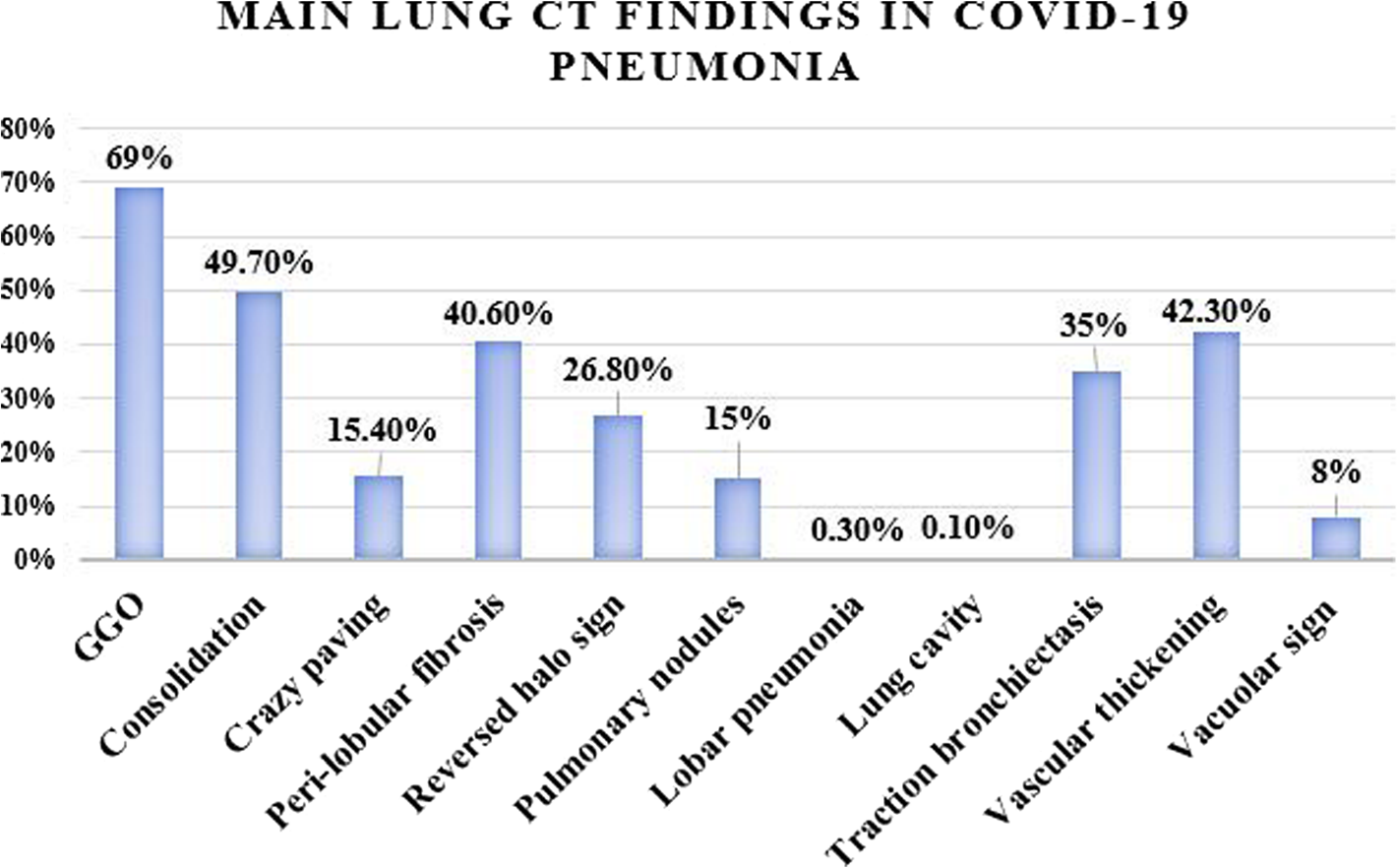
Bar chart showing the prevalence of the main lung parenchymal CT findings in COVID-19 pneumonia

Male participants were more than females in both the positive (304/569, 53.4%) and negative (127/203, 62.6%) groups; however, it was not a statistically significant difference (*p* value = 0.058) (Fig. 2).

**Fig. 3:**
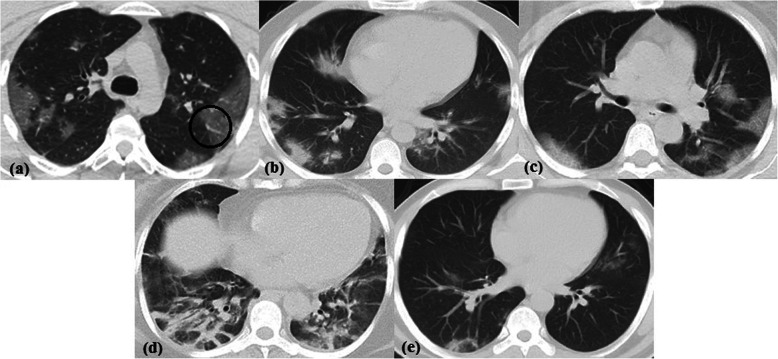
Non-contrast CT chest axial cuts in lung window showing the main findings in RT-PCR proved COVID-19 cases. **a** Multifocal bilateral peripheral GGO in a 35-year-old male associated with vascular thickening (circle). **b** Multifocal bilateral peripheral consolidation in a 45-year-old male. **c** Multifocal bilateral peripheral and central crazy paving in a 55-year-old female. **d** Peri-lobular fibrosis in a 50-year-old female. **e** Right lower lung lobe posterior segment reversed halo sign in a 46-year-old male

**Fig. 4:**
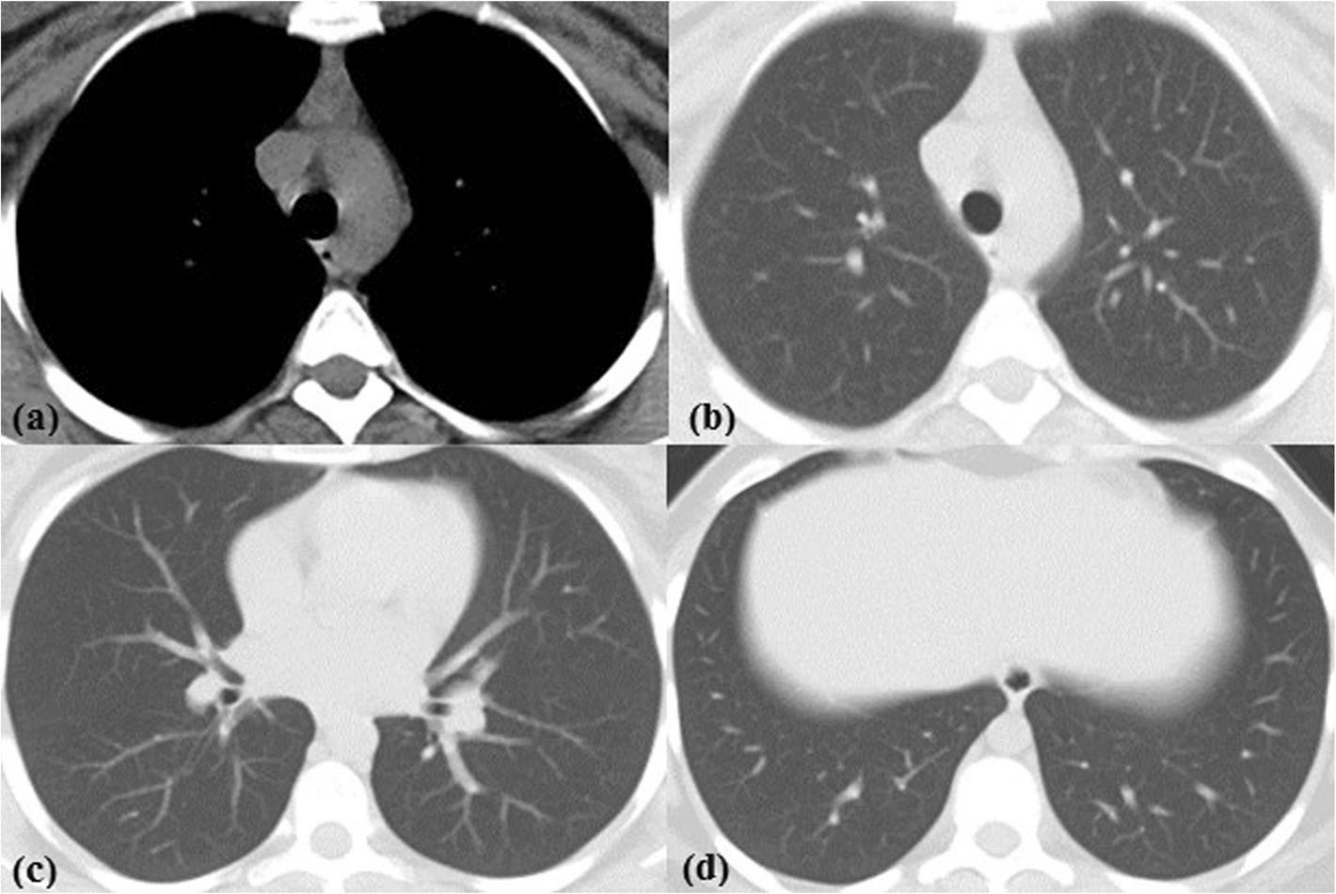
Thymic hyperplasia in a 23-year-old female with RT-PCR proved COVID-19. **a** Non-contrast CT chest axial cut mediastinal window shows retrosternal well-defined triangular-shaped soft tissue density with straight borders with less macroscopic fat than expected for age. **b–d** Axial lung window showing no lung abnormality

Excluding 225/862 (26.1%) normal chest CT studies, the imaging findings of the 637/862 (73.9%) positive CT studies included ground glass opacities in 595/862 (69%), consolidation in 428/862 (49.7%), crazy paving in 133/862 (15.4%), peri-lobular fibrosis in 350/862 (40.6%), reversed halo sign in 231/862 (26.8%), ground glass or sub-solid peripheral pulmonary nodules in 129/862 (15%), lobar pneumonia in 3/862 (0.3%) studies, and lung cavity in 1/862 (0.1%) study only (Fig. 3). Traction bronchiectasis was also observed in 302/862 (35%), vascular thickening in 365/862 (42.3%), and vacuolar sign in 69/862 (8%) studies. Associated finding of pleural thickening was detected in 105/862 (12.2%) studies; pleural effusion in 27/862 (3.1%) studies; 22/862 (2.6%) bilateral, 4/862 (0.5%) on the left side, and 1/862 (0.1%) on the right side; pericardial effusion in 39/862 (4.5%) studies; lymphadenopathy in 47/862 (5.5%) studies; 10/862 (1.2%) non-calcified and 37/862 (4.3%) calcified; and lung cysts in 7/862 (0.8%) studies. Thymic hyperplasia [defined as retrosternal well-defined triangular-shaped soft tissue density with straight borders showing less macroscopic fat than expected for age [9] was identified in 123/862 (14.3%) of studies (42 males and 81 females, age range 17–50 years; mean 26.1 years); out of which 102/862 (11.8%) (37 males and 65 females, age range 21–50 years; mean 25.98 years) showed no CT lung findings of COVID-19 pneumonia with a statistically significant predilection for young females (Fig. 4). The prevalence of common and associated CT findings is reported in Tables 2 and 3 and Fig. 2.

**Table 2:** Main CT imaging findings in COVID-19 pneumonia

Imaging finding	No. of patients (%)
Ground glass opacities	595 (69%)
Consolidation	428 (49.7%)
Crazy paving	133 (15.4%)
Peri-lobular fibrosis	350 (40.6%)
Reversed halo sign	231 (26.8%)
Peripheral subsolid pulmonary nodules	129 (15%)
Lobar pneumonia	3 (0.3%)
Lung cavity	1 (0.1%)
Traction bronchiectasis	302 (35%)
Vascular thickening	365 (42.3%)
Vacuolar sign	69 (8%)

**Table 3:** Associated CT imaging findings in COVID-19 pneumonia

Imaging finding	No. of patients (%)
**Pulmonary**	
Old granulomatous infection (apical scarring/calcified nodules)	73 (8.47%)
Cysts	7 (0.8%)
Airway disease	92 (10.67%)
**Mediastinal**	
Lymphadenopathy: calcified/noncalcified	47 (5.5%)/37 (4.3%)/10 (1.2%)
Pericardial effusion	39 (4.52%)
Cardiomegaly	123 (14.27%)
Pulmonary hypertension	42 (4.87%)
Anterior mediastinal mass	1 (0.12%)
Mediastinal bronchogenic cyst	1 (0.12%)
Tracheal diverticulum	5 (0.58%)
Thymic hyperplasia	123 (14.3%)
Atheromatous vascular plaques	111 (12.88%)
Ectatic ascending aorta	17 (1.97%)
Lipomatosis	1 (0.12%)
Median sternotomy sutures	3 (0.35%)
Enlarged heterogeneous thyroid gland	48 (5.57%)
Patulous esophagus	3 (0.35%)
**Pleural**	
Effusion	27 (3.1%)
Thickening/reaction	105 (12.2%)
Calcified plaques (Asbestos exposure)	2 (0.23%)
**Chest wall**	
Breast mass	4 (0.46%)
**Abdominal**	
Liver cirrhosis	17 (1.97%)
Fatty liver	69 (8%)
Hepatomegaly/Splenomegaly	14 (1.62%)/13 (1.51%)
Hepatic/Splenic calcified focus	9 (1%)
Hepatic/splenic focal lesion	9 (1%)
Adrenal mass	10 (1.16%)
Renal stone and backpressure changes	21 (2.4%)
Renal cortical cyst	14 (1.62%)
Renal atrophic changes	3 (0.35%)
Ascites	1 (0.12%)
Calcular gall bladder	26 (3.02%)

Both lungs were involved in 588/862 (68.2%), the right lung in 622/862 (72.2%), and the left lung in 603/862 (70%) studies; the right upper lobe was implicated in 532/862 (61.7%), middle lobe in 504/862 (58.5%), right lower lobe in 609/862 (70.6%), left upper lobe in 528/862 (61.3%), lingula in 489/862 (56.7%), and left lower lobe in 578/862 (67.1%) studies. Unilateral lung affection was encountered in 49/862 (5.7%) of studies while uni-lobar affection in 68/862 (7.9%). The lesions showed peripheral distribution in 624/862 (72.4%), both peripheral and central in 222/862 (25.8%), patchy in 558/862 (64.7%), and diffuse in 16/862 (1.9%) studies. The involved lung lobes and the lesions’ distribution are recoded in Table 4.

**Table 4:** Distribution of Lesions in COVID-19 pneumonia patients

Category and subcategory	No. of Patients (%)
Involved lungs and lobes	
Both lungs	588 (68.2%)
Right lung	622 (72.2%)
Left lung	603 (70%)
Right upper lobe	532 (61.7%)
Middle lobe	504 (58.5%)
Right lower lobe	609 (70.6%)
Left upper lobe	528 (61.3%)
Lingula	489 (56.7%)
Left lower lobe	578 (67.1%)
Lesion distribution	
Peripheral	626 (72.6%)
Peripheral and central	222 (25.8%)
Patchy	558 (64.7%)
Diffuse	16 (1.9%)

According to the four categories endorsed by the RSNA Expert Consensus Statement, the CT findings in our study were reported as typical in 594/862 (68.9%), indeterminate in 31/862 (3.6%), and atypical in 12/862 (1.4%) studies, while chest CT was normal; thus, reported as negative for COVID-19 pneumonia in 225/862 (26.1%) studies.

**Fig. 5:**
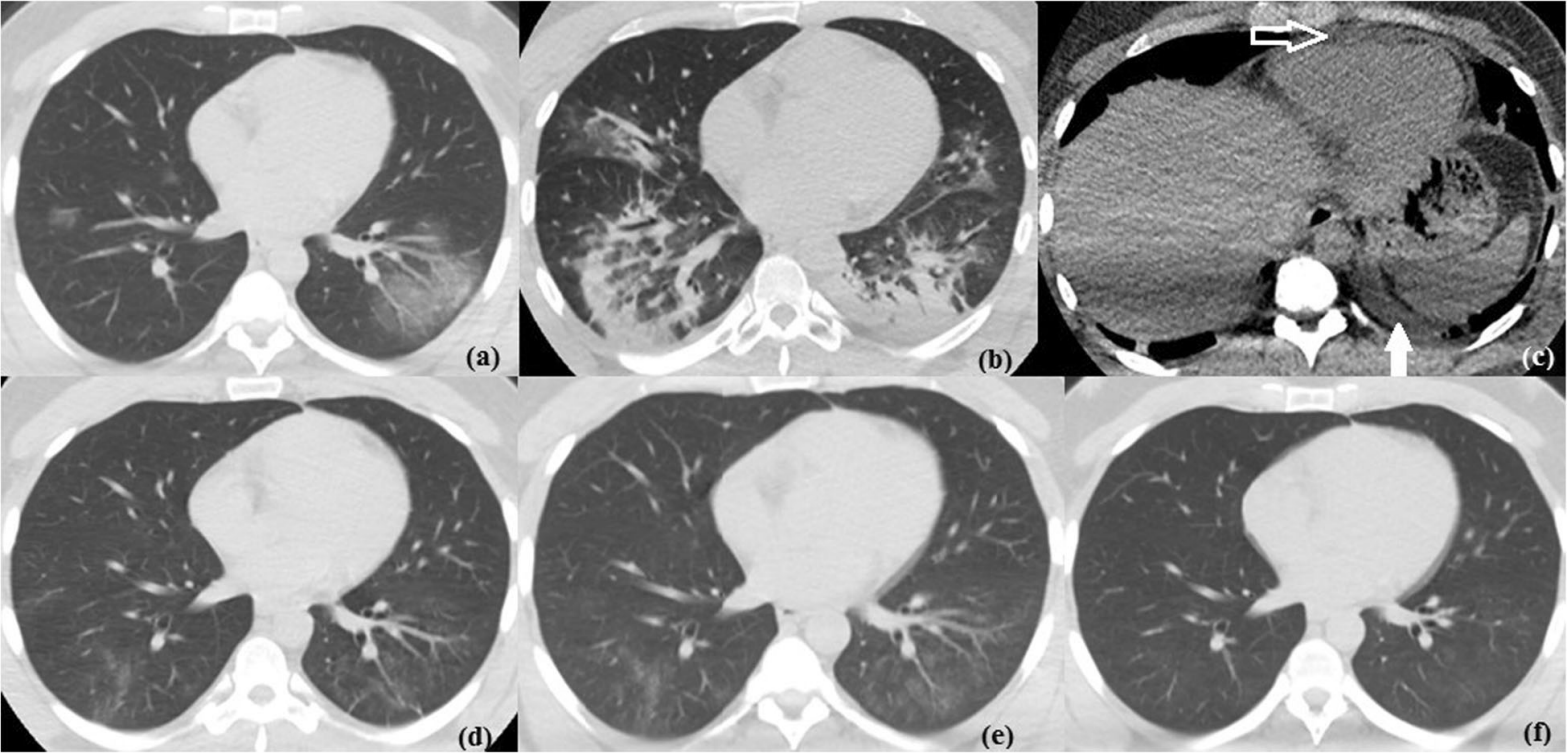
Temporal CT changes in a 35-year-old male with COVID-19. **a** Initial non-contrast CT chest axial lung window showing multifocal bilateral predominantly peripheral patchy ground glass opacities with vascular thickening. **b**, **c** Follow up CT study after 7 days in axial lung and axial non-contrast mediastinal windows showing progressive course regarding the extent of lung involvement with bilateral predominantly peripheral patchy consolidation and air bronchogram associated with newly developed mild pericardial effusion (hollow arrow) and mild left pleural effusion (solid arrow). **d**–**f** Follow up images 16, 22, and 28 days respectively after initial CT showing gradual regressive course with only ill-defined faint GGO seen

Seventy-one participants underwent 90 follow-up CT studies according to clinical context (Fig. 5). Fifty-six performed 1 follow up, twelve performed 2 follow ups, two performed 3 follow ups, and one performed 4 follow ups. The time interval between the scans ranged from 2 to 49 days, with an average of 13.2 days.

Twenty-one participants showing normal initial CT underwent follow-up CT studies. One of them established imaging features of COVID-19 6 days after the initial CT, while the other 20/72 (27.8%) (age range 21–53 years; mean 32.65 years) had their follow up studies guided by a persistent positive RT-PCR at time interval ranging between 4 and 49 days from the initial to the follow up CT; yet, their chest CT studies remained normal on follow up.

The follow-up CT studies showed progression in 17/90 (18.9%), regression in 44/90 (48.9%), and stationary course in 29/90 (32.2%) studies.

## Discussion

COVID-19 pandemic has affected millions of people worldwide. Thorough comprehension of CT imaging features of COVID-19 is mandatory for effective patient management. Hence, we meticulously assessed the CT studies of 772 participants with RT-PCR proved COVID-19.

About one-quarter of them, with statistically significant younger age, showed normal initial CT study. This is consistent with the previous studies done by Ojha et al., Yang et al., Asefi and Safaie Xu et al., and Zhang et al. [6, 10–13]. They stated that up to half of patients with positive RT-PCR may show a normal CT especially in the early phase or in asymptomatic infections. Therefore, CT cannot completely exclude COVID-19 infection.

In conformity with nearly all published studies [6, 7, 11, 13–25], the most prevailing CT imaging finding of COVID-19 in our population was GGO with or without consolidation/crazy paving followed by peri-lobular fibrosis combined with vascular thickening and bronchiectasis in a bilateral predominantly peripheral distribution with lower lobe predilection. On the other hand, lobar pneumonia and lung cavitation as well as diffuse, unilateral, or unifocal distributions were uncommon with the middle lobe and lingula being the least affected.

Similar to Carotti et al. [7], the most common associated imaging finding in COVID-19 was minimal lower lobar posterior pleural thickening.

In accordance with Salehi et al. and Kim et al. [14, 26], pleural effusion, pericardial effusion, and mediastinal lymphadenopathy were infrequently described associated findings.

It is worth noting that pleural and/or pericardial effusion were particularly reported in cases with advanced lung affection in the form of extensive multi-lobar consolidation and/or crazy paving. This agrees with Ojha et al.’s [6] declaration that pleural and pericardial effusion occur in advanced cases.

More than two-thirds of the mediastinal lymph nodes reported in our study were calcified, likely being a sequel of healed previous granulomatous infection. This may be attributed to the fact that tuberculosis is endemic in Egypt.

In few of our cases, a lung cyst was recognized among the other CT findings. A limited number of articles mentioned the presence of cyst(s) in cases of COVID-19; however, the etiology and relation to COVID-19 pneumonia are unclear [19, 22].

On searching the literature, none of the previous studies described thymic abnormalities in association with COVID-19. Nevertheless, thymic hyperplasia was identified in a considerable number of our cases with statistically significant young age and female predilection; most of them had no lung abnormalities on CT. The thymus is a lymphoid organ that plays a cardinal role in development of the immune system during childhood. It gradually involutes throughout maturation yet maintains the ability to re-grow (9). Hence, we suggest that thymic hyperplasia is an immune response to the viral infection; yet, further studies are warranted to validate this hypothesis.

Employing the previously illustrated RSNA Expert Consensus Statement, typical category was the most frequently encountered, while indeterminate and atypical categories were unusual. Correspondingly, CT can confidently diagnose COVID-19 in about 69% of cases. Taking into consideration the current pandemic and resources constraints (e.g., RT-PCR availability), the implementation of CT as a screening tool can be disputed.

Regarding follow up, progression was defined as an increase in the number, size, extent, or density of previously noted lesion(s) and/or development of new lesions, while regression would represent decrease in those finding(s). In this study, nearly half of the evaluated studies showed regression, one-third remained unchanged, and few cases showed progression.

On reviewing previously published studies, the percentage of cases with progressive CT findings ranged from 32 to 94.75%. This wide range may be explained by the variability in study duration, number of cases, and time interval between initial and follow up CT studies [13, 14, 16, 17, 19, 20, 27].

Twenty of our cases had persistent normal CT on follow up. In Xu et al.’s study [12], 75% of cases had normal initial and follow up scans. Also, 13% of patients evaluated by Zhang et al. [13] and 1.8% of those assessed by Guan et al. [16] remained negative on follow up. Consequently, we suggest that patients may not develop CT manifestations of pneumonia along the course of COVID-19 infection.

One of the major strengths of this study is its timing; as it was conducted early along the course of this not yet fully understood pandemic. Thereby, the whole radiological spectrum of COVID-19 was captured making this one of the most sensitive radiological studies of COVID-19 cases in Egypt.

However, our study had some limitations including the unavailability of laboratory data and limited number of follow up studies. Furthermore, this is a hospital not a population-based study; therefore, sensitivity and specificity could not be calculated; yet, we could deduct the percentage of different CT imaging categories. It is thus recommended to perform future studies to confirm the generalizability of this study and similar ones.

## Conclusion

In conclusion, although COVID-19 cannot be entirely excluded by chest CT, it can be efficiently distinguished in more than two-thirds of cases; making CT a universally available, non-invasive, and rapid diagnostic tool for COVID-19.

## Data Availability

The datasets used and/or analyzed during the current study are available from the corresponding author on reasonable request.

## Abbreviations

COVID-19: Coronavirus Disease 2019
GGO: Ground glass opacities
IRB: Institutional review board
NAT: Nucleic acid testing
RSNA: Radiological Society of North America
RT-PCR: Reverse-transcriptase polymerase-chain-reaction
SARS-CoV-2: Severe acute respiratory syndrome coronavirus 2
SPSS: Statistical Product and Service Solutions
WHO: World Health Organization
